# Cancer of Unknown Primary Presenting as Isolated FDG-Avid Cervical Lymph Node Metastasis: Diagnostic Escalation after a Non-Diagnostic Core Biopsy – A Case Report

**DOI:** 10.1159/cro/abzag035

**Published:** 2026-08-27

**Authors:** Dorota Szydlarska, Zuzanna Banaszkiewicz, Małgorzata Kołos, Alicja Jakubowska, Katarzyna Życińska

**Affiliations:** a Department of Digital Medicine, Implementation and Innovation, National Medical Institute of the Ministry of the Interior and Administration, Warsaw, Poland; b Department of Family Medicine, Medical University of Warsaw, Warsaw, Poland; c National Medical Institute of the Ministry of the Interior and Administration, Warsaw, Poland; d Department of Rheumatology, Connective Tissue Diseases, and Rare Diseases, National Medical Institute of the Ministry of the Interior and Administration, Warsaw, Poland

**Keywords:** Cancer of unknown primary, Cervical lymph node metastasis, Excisional biopsy, 18F-Fluorodeoxyglucose positron emission tomography/computed tomography

## Abstract

**Introduction:**

Cancer of unknown primary (CUP) remains a diagnostic and therapeutic challenge, particularly when presenting as isolated cervical lymph node disease. Adequate tissue sampling is crucial, and escalation to excisional biopsy may be necessary when minimally invasive procedures are inconclusive.

**Case Presentation:**

A 62-year-old man with a history of long-term tobacco use presented with a right-sided neck mass. Otolaryngologic examination and fiberoscopy revealed no mucosal abnormalities. Fine-needle aspiration suggested metastatic poorly differentiated carcinoma, while core needle biopsy yielded scant, nondiagnostic material. Contrast-enhanced CT demonstrated pathological right cervical lymph nodes (level III 29 × 29 mm, level IIa 20 × 15 mm). 18F-Fluorodeoxyglucose positron emission tomography/computed tomography showed an FDG-avid right cervical nodal package at the level IIB/III border (SUVmax 21.1) and an incidental focal uptake in the left thyroid lobe (SUVmax 4.4), with no other suspicious lesions. Thyroid ultrasound revealed a small, well-demarcated hypoechoic lesion (3.5 × 2.6 × 4.5 mm); repeated ultrasound-guided FNAC was benign (Bethesda II), and calcitonin was within the reference range. The patient underwent excisional lymph node biopsy and comprehensive endoscopic evaluation under general anesthesia (panendoscopy with bilateral tonsillectomy and directed biopsies), without identification of a primary tumor. Histopathology and immunohistochemistry confirmed nodal metastasis of undifferentiated carcinoma, most consistent with carcinoma with sarcomatoid features, confined within the lymph node capsule. Because no primary site was identified after imaging, endoscopic evaluation, thyroid workup, and histopathological assessment of the lymph node, the diagnosis remained CUP. The patient was subsequently referred for radiotherapy.

**Conclusion:**

In cervical lymph node metastasis from CUP, adequate tissue sampling remains essential for definitive diagnosis. When fine-needle aspiration suggests malignancy but core needle biopsy is nondiagnostic, early excisional biopsy should be considered to enable reliable histopathological and immunohistochemical classification. PET/CT may guide the diagnostic pathway and identify incidental findings requiring verification, but imaging should not delay escalation to representative tissue sampling.

## Introduction

Cancer of unknown primary (CUP) is defined by cytologically or histologically confirmed metastasis in which the primary site cannot be identified despite standard diagnostic workup, including clinical assessment, laboratory testing, imaging, and pathological evaluation [1]. Patients with a single metastatic focus may be candidates for potentially curative local therapy, such as surgical excision of the metastasis, targeted radiotherapy, or combined modalities, depending on metastatic site and clinical context [1].

In suspected CUP, the initial evaluation typically includes thorough history and physical examination, baseline laboratory tests (including complete blood count, electrolytes, renal and hepatic function, LDH, urinalysis, fecal occult blood test), and imaging such as CT of the chest, abdomen, and pelvis, as well as selected endoscopic examinations based on clinical indications [1]. Routine assessment of a broad panel of serum tumor markers is generally discouraged due to limited specificity, although selected markers may be useful in appropriate settings [1].

Cervical lymph node metastasis without an identifiable mucosal primary warrants particular attention because of the implications for head and neck-directed diagnostics and treatment. In this setting, the central diagnostic challenge is often not imaging alone but the adequacy of tissue sampling needed to establish a reliable clinicopathological diagnosis [1, 2].

Imaging plays a pivotal supportive role in the diagnostic workup of CUP, particularly in patients presenting with isolated cervical lymphadenopathy. Cross-sectional imaging and 18F-fluorodeoxyglucose positron emission tomography/computed tomography (18F-FDG PET/CT) help detect occult primary sites, stage disease, guide biopsy, and identify incidental findings that require histological verification. However, even advanced imaging techniques cannot replace adequate histopathological sampling when the diagnosis remains uncertain.

## Case Report

A 62-year-old man with a history of long-term tobacco use and well-controlled arterial hypertension treated with antihypertensive medication presented to a primary care physician due to a progressive increase in right-sided neck circumference. No other abnormalities were noted on history or physical examination. Baseline biochemical tests and selected tumor markers were within reference ranges, including PSA, calcitonin, CA19-9, AFP, CEA, and LDH (Table 1).

**Table 1.** Selected laboratory tests and tumor markers

Analyte (reference range)	Result
WBC 4.5–10.0 × 10^3^/μL	9.01
RBC 4.7–6.1 × 10^6^/μL	4.8
Hb 14.0–17.0 g/dL	14.6
Plt 150–400 × 10^3^/μL	258
INR 0.9–1.2	0.95
Na 134–145 mmol/L	141
K 3.5–5.3 mmol/L	5.1
Glucose 70–99 mg/dL	99
ESR 0–20 mm/h	1.0
CRP <5 mg/L	1.3
Urea 17–49 mg/dL	36
Creatinine 0.7–1.25 mg/dL	0.9
PSA 0.0–4.0 ng/mL	0.53
Calcitonin 0.0–8.4 pg/mL	7.1
CA 19–9 0.0–37.0 U/mL	5.3
AFP 0.89–8.78 ng/mL	2.12
CEA 0.0–5.0 ng/mL	1.9
LDH 125–243 U/L	165.0

WBC, white blood cell count; RBC, red blood cell count; Hb, hemoglobin; Plt, platelet count; INR, international normalized ratio; Na, sodium; K, potassium; ESR, erythrocyte sedimentation rate; CRP, C-reactive protein; PSA, pro-state-specific antigen; CA 19-9, carbohydrate antigen 19-9; AFP, alpha-fetoprotein; CEA, carcinoembryonic antigen; LDH, lactate dehydrogenase.

Otolaryngologic consultation revealed a firm nodal package at the border of neck levels II–III on the right side. Examination of the oral cavity, pharynx, nasal cavity, nasopharynx, and larynx was unremarkable, and fiberoscopy showed no abnormalities. Ultrasound-guided fine-needle aspiration cytology of the cervical lymph node was consistent with metastatic malignancy, initially described as a poorly differentiated carcinoma (most likely glandular). Abdominal ultrasound showed no significant abnormalities. Upper and lower gastrointestinal endoscopy (gastroscopy and colonoscopy) revealed no pathology.

Contrast-enhanced CT of the neck and chest demonstrated pathological right cervical lymph nodes: level III 29 × 29 mm deforming the right internal jugular vein and level IIa 20 × 15 mm. Tonsilloliths up to approximately 5 mm were noted bilaterally; no other significant findings were reported. Core needle biopsy yielded scant material consisting of small fragments of lymphoid tissue with fibrosis; immunophenotyping revealed mixed T (CD3) and B (CD20) lymphocytes, CD23 in a dendritic cell network, and sparse CD30-positive cells. The specimen was considered of low diagnostic value. MRI of the neck was not performed because contrast-enhanced CT combined with PET/CT was considered sufficient for anatomical and metabolic assessment, and multidisciplinary evaluation did not identify an additional diagnostic benefit of MRI.

18F-FDG PET/CT demonstrated an FDG-avid right cervical nodal package located at the border of levels IIB/III (SUVmax 21.1) (Fig. 1). According to the official PET/CT report, focal FDG uptake was also identified in the left thyroid lobe (SUVmax 4.4), prompting further evaluation. No other lesions with increased tracer uptake were found, including no findings suggestive of the primary tumor site. The apparent discrepancy between CT and PET/CT findings reflects different characterization of the same nodal disease. CT identified two enlarged lymph nodes in levels IIa and III, whereas PET/CT demonstrated intense FDG uptake within a metabolically active nodal package extending across levels IIB and III. No abnormal FDG uptake was identified within the palatine tonsils or other mucosal structures of the upper aerodigestive tract.

**Fig. 1.**
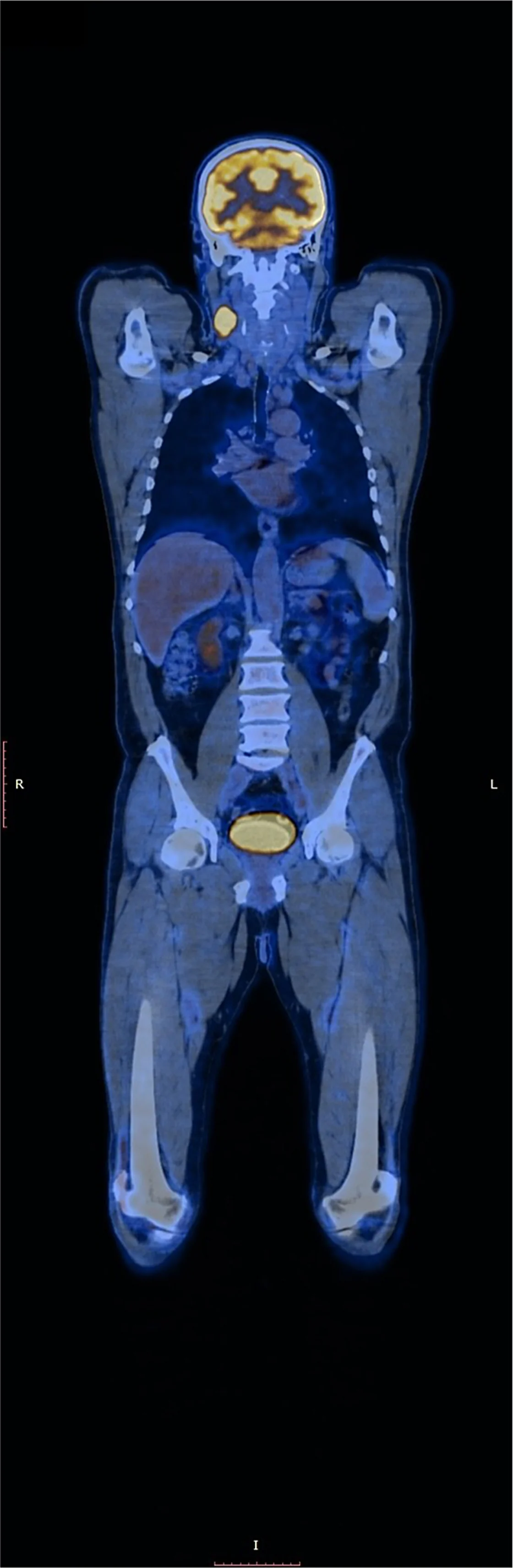
18F-FDG PET/CT image demonstrating intense FDG uptake within the right cervical nodal package at the border of levels IIB/III (SUVmax 21.1). According to the official PET/CT report, focal FDG uptake was also identified in the left thyroid lobe (SUVmax 4.4), prompting further thyroid evaluation by dedicated ultrasound and fine-needle aspiration cytology. FDG, fluorodeoxyglucose; PET/CT, positron emission tomography/computed tomography; SUVmax, maximum standardized uptake value.

Thyroid ultrasound revealed a well-demarcated solid hypoechoic focal lesion adjacent to the anterior wall of the left thyroid lobe, measuring approximately 3.5 × 2.6 mm in transverse view, with a maximal longitudinal dimension of approximately 4.5 mm (Fig. 2a, b). Ultrasound-guided FNAC of the thyroid lesion was benign (Bethesda category II). Repeat thyroid FNAC also remained benign, and the normal calcitonin level provided no support for medullary thyroid carcinoma.

**Fig. 2.**
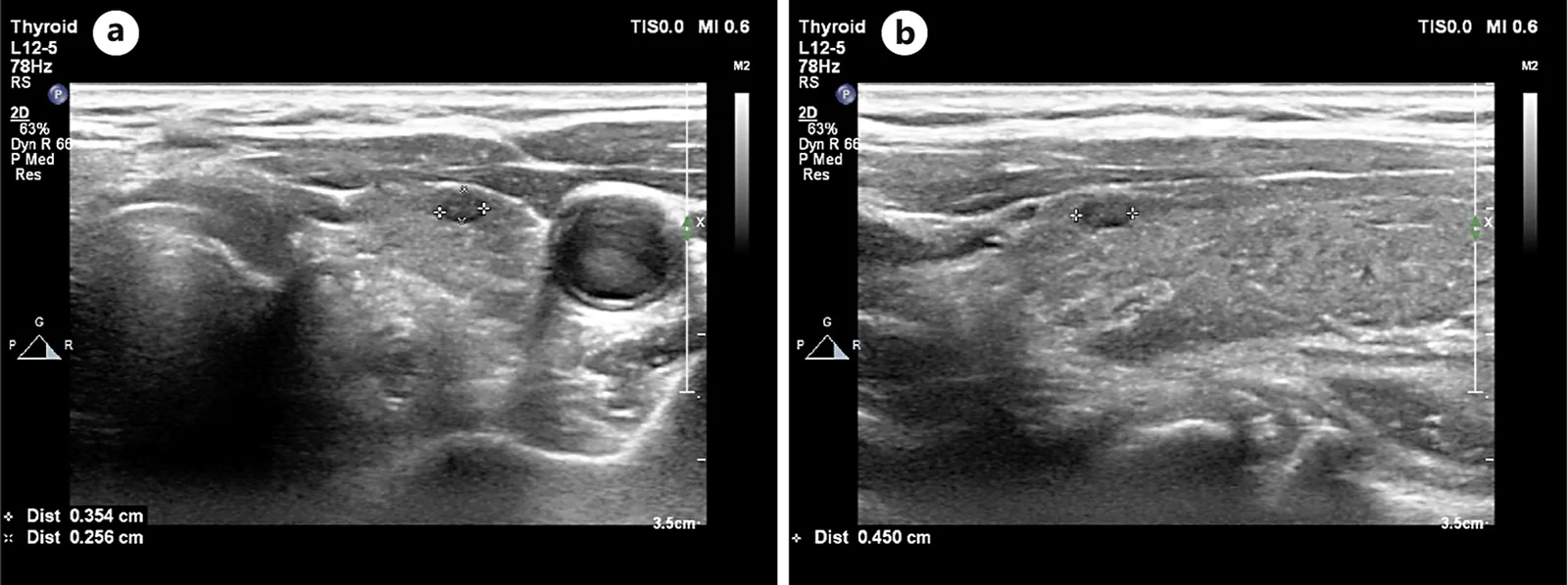
Thyroid ultrasound. **a** Transverse view showing a well-demarcated solid hypoechoic focal lesion adjacent to the anterior wall of the left thyroid lobe, measuring approximately 3.5 × 2.6 mm. **b** Longitudinal view of the same lesion showing a maximal dimension of approximately 4.5 mm.

The diagnostic sequence was therefore interpreted as follows: metastatic malignancy was confirmed cytologically in the cervical lymph node; common clinically relevant primary sites were assessed by ENT examination with fiberoscopy, CT of the neck and chest, abdominal ultrasound, upper and lower gastrointestinal endoscopy, 18F-FDG PET/CT, thyroid ultrasound and FNAC; and no primary site was identified. Given the absence of an identifiable primary tumor and the nondiagnostic core biopsy, the patient was referred for surgical management. After appropriate preparation, excisional biopsy of the right cervical lymph node was performed along with panendoscopy under general anesthesia, including bilateral tonsillectomy and directed biopsies of the pharynx, larynx, esophageal inlet, and nasopharynx. Histopathological findings are presented below (Fig. 3a–e).

**Fig. 3.**
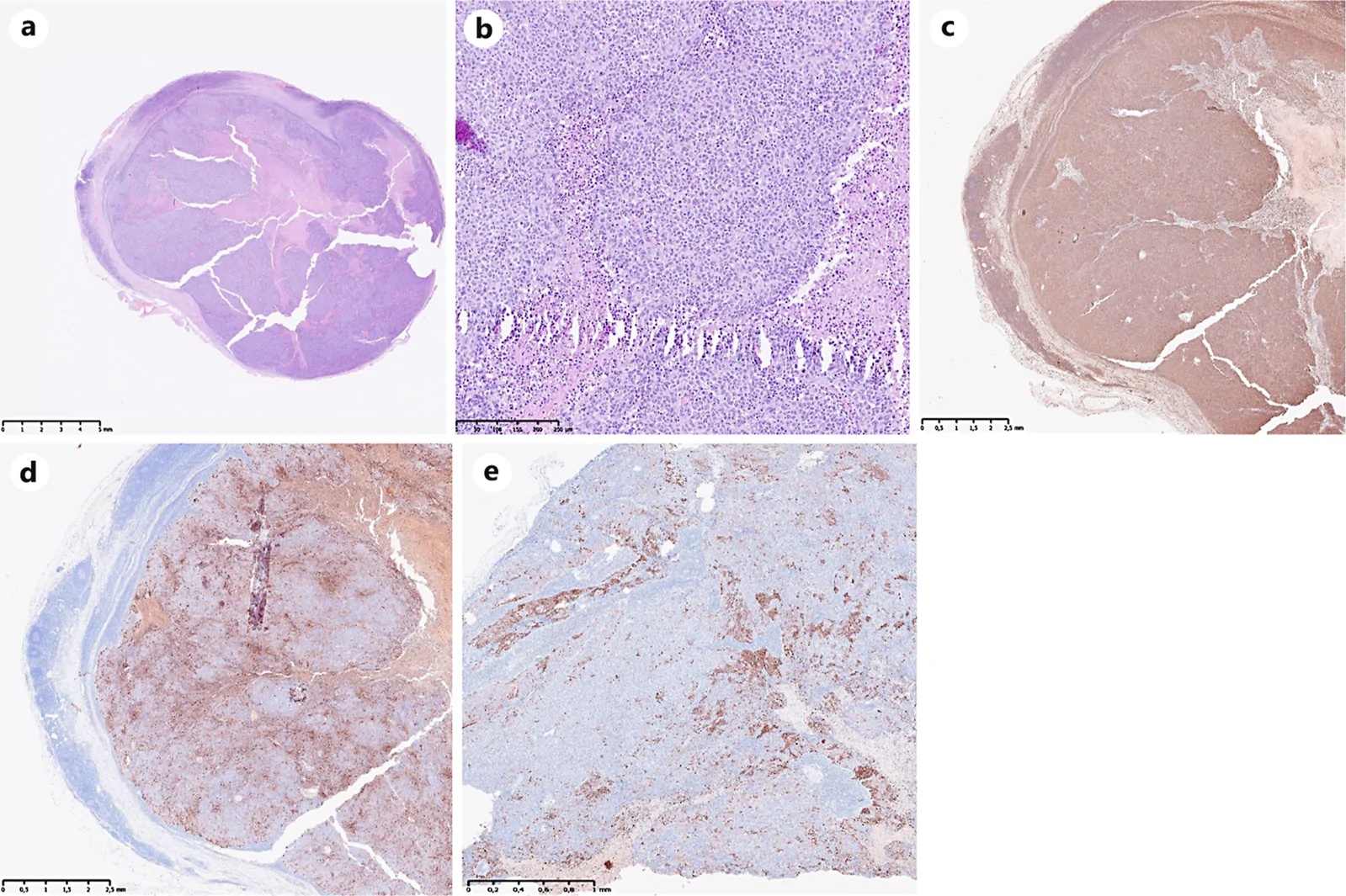
Histopathology of the excised cervical lymph node, obtained from scanned whole-slide preparations; scale bars are shown in each panel. **a** H&E staining showing lymph node architecture infiltrated by a poorly differentiated malignant tumor. **b** H&E staining, higher-resolution view, showing atypical epithelioid tumor cells without organoid structures and with central necrosis. **c** Immunohistochemistry: vimentin-positive reaction in tumor cells. **d** Immunohistochemistry: EMA-focal positivity in tumor cells. **e** Immunohistochemistry: cytokeratin AE1/AE3-focal positivity in tumor cells.

Histopathology: macroscopic examination of the excised lymph node revealed an encapsulated, firm, whitish nodule measuring 3.2 × 2.5 × 1.7 cm. Microscopy showed metastasis of a poorly differentiated malignant tumor confined to the lymph node, without extension beyond the capsule (no extranodal extension) (Fig. 3a, b). Immunohistochemical staining demonstrated diffuse vimentin positivity with focal expression of EMA and cytokeratin AE1/AE3 (Fig. 3c–e). Tumor cells were negative for p40, p16, EBER, S-100, CD45 (LCA), CD56, chromogranin A, CD34, and SOX10; synaptophysin showed a likely nonspecific reaction. INI1/SMARCB1 immunohistochemical staining was not performed and was not available in the initial diagnostic panel. Therefore, reference-center consultation with additional immunohistochemical and molecular testing, including INI1/SMARCB1 assessment, was recommended to further exclude rare entities such as SMARCB1-deficient sinonasal carcinoma and epithelioid sarcoma. Based on morphology and immunoprofile, the findings favored metastasis of an undifferentiated carcinoma, most likely carcinoma with sarcomatoid features. Palatine tonsils showed reactive lymphoid hyperplasia and focal fibrosis with *Actinomyces* colonies in the crypts; no malignancy was identified. The combined epithelial marker expression with diffuse vimentin positivity and absence of lineage-specific markers argued against melanoma, lymphoma, and true mesenchymal malignancy.

The patient subsequently underwent follow-up PET/CT, which again did not reveal any hypermetabolic lesion other than the left thyroid lobe; repeated thyroid FNAC remained benign (Bethesda category II). In the continued absence of an identifiable primary site, the patient was referred for radiotherapy. The stepwise diagnostic pathway is summarized in Figure 4.

**Fig. 4.**
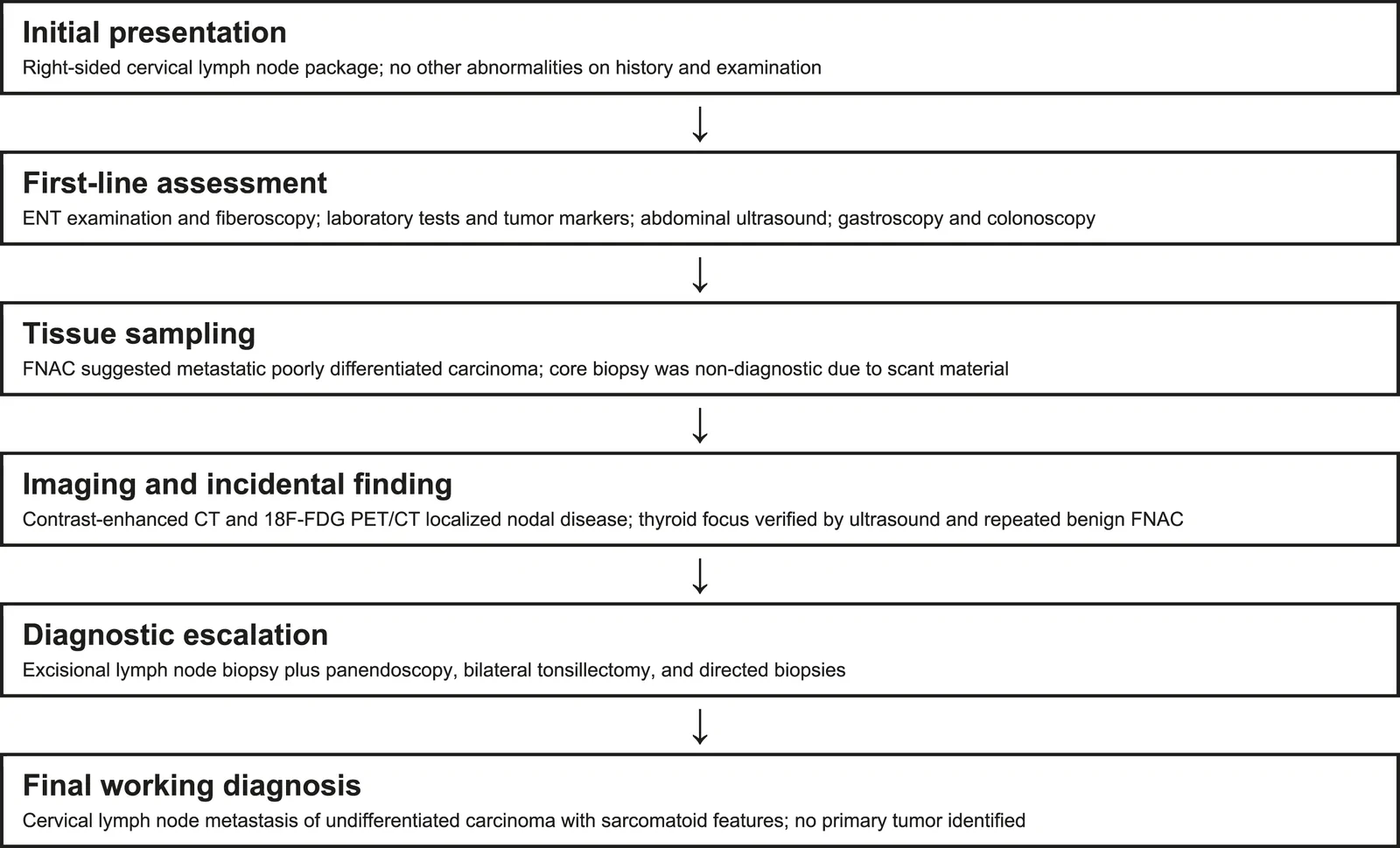
Structured diagnostic pathway in the reported case, summarizing the stepwise exclusion of major clinically relevant primary sites and the escalation from non-diagnostic minimally invasive biopsy to excisional lymph node biopsy. H&E, hematoxylin and eosin; EMA, epithelial membrane antigen.

## Discussion

This case highlights the diagnostic limitations of minimally invasive tissue sampling in selected patients with cervical lymph node metastasis from CUP. Despite extensive imaging, PET/CT evaluation, and comprehensive endoscopic assessment, definitive diagnosis was achieved only after complete excision of the involved lymph node. The principal value of this report lies not in the rarity of CUP itself but in demonstrating the importance of timely diagnostic escalation after a nondiagnostic core needle biopsy.

CUP constitutes a heterogeneous group of malignancies in which the primary site remains unidentified despite standard diagnostics. Polish recommendations estimate that CUP accounts for approximately 3% of all malignancies and emphasize that precise pathological characterization of the metastatic lesion is pivotal for directing further diagnostics and treatment [1]. European guidelines similarly indicate that CUP accounts for <5% of oncologic diagnoses and that this proportion is decreasing with advances in imaging and pathology; nevertheless, in many patients the primary tumor is never identified throughout the disease course [2].

The central learning point of this case is that diagnostic escalation should not be delayed when minimally invasive sampling is inconclusive. Isolated right cervical lymph node involvement (levels II–III) was the only manifestation of disease, which mandated a head and neck-focused diagnostic pathway and representative tissue for accurate classification. Guidelines and expert recommendations highlight that, in poorly differentiated metastases, adequate tissue may require repeat sampling, including excisional biopsy, and broad immunohistochemical assessment [1, 2]. In this patient, core needle biopsy was nondiagnostic, whereas excisional biopsy enabled classification as undifferentiated carcinoma with sarcomatoid features. Thus, the diagnostic yield and reliability depended primarily on tissue quantity and quality rather than on additional imaging alone [1, 2].

The differential diagnosis was approached systematically. A mucosal head and neck primary was considered because of the cervical nodal location and the patient’s tobacco exposure; it was not identified on ENT examination, fiberoscopy, panendoscopy, bilateral tonsillectomy, or directed biopsies [3, 4]. The diagnostic value of tonsillectomy in cervical nodal metastasis of unknown primary has also been discussed in relation to p16 status, although in the present case p16 immunohistochemistry was negative [5]. Importantly, no abnormal FDG uptake was observed within the palatine tonsils on PET/CT. Nevertheless, bilateral tonsillectomy was performed because occult oropharyngeal primaries may remain PET negative and can only be excluded by histopathological examination. Pulmonary or other thoracic primaries were not supported by CT of the chest or PET/CT. Gastrointestinal malignancy was not supported by gastroscopy, colonoscopy, abdominal ultrasound, tumor markers, or PET/CT. The incidental thyroid focus required particular caution: its SUVmax was 4.4, ultrasound showed a small well-demarcated hypoechoic lesion, repeated FNAC was benign (Bethesda II), and calcitonin was normal, supporting interpretation as incidental rather than the source of metastasis. No thyroid surgical specimen was available, and thyroid-specific immunohistochemistry to exclude anaplastic thyroid carcinoma was therefore not performed; this has been acknowledged as a limitation. Lymphoma, melanoma, and sarcoma were considered unlikely because of the available immunophenotype: tumor cells were negative for CD45, S-100, SOX10, and CD34, while focal epithelial marker expression with diffuse vimentin positivity favored carcinoma with sarcomatoid features.

For cervical node metastasis in CUP, 18F-FDG PET/CT remains useful to identify lesions warranting targeted biopsy and to search for thoracic primaries or additional metastases, while acknowledging false-positive incidental findings [1]. Observational data in cohorts with cervical nodal metastases in CUP suggest that PET/CT may reveal suspicious foci in a substantial fraction of patients and provides relatively high specificity, but negative results do not close diagnostics and positive findings require histologic verification [6]. In this case, PET/CT localized metabolically active nodal disease and detected an incidental thyroid focus; however, the decisive diagnostic step was excisional biopsy of the cervical lymph node.

Strengths and limitations: a major strength of the diagnostic process was its stepwise, guideline-concordant escalation: cross-sectional imaging including PET/CT, endoscopic evaluation under general anesthesia with biopsies, and excisional biopsy after a nondiagnostic core biopsy [1–3]. The key limitation – typical for CUP – remains failure to identify the primary site despite extensive workup [2]. Another limitation is that INI1/SMARCB1 immunohistochemistry, thyroid-specific immunohistochemistry, and molecular testing were not available in the initial workup; these tests may be useful in reference-center review to further refine the differential diagnosis, including SMARCB1-deficient sinonasal carcinoma, epithelioid sarcoma, and rare thyroid-derived malignancies.

### Clinical Implications

This case underlines that in cervical CUP, minimally invasive biopsies may be insufficient in poorly differentiated tumors and should not delay excisional biopsy when diagnostic uncertainty persists. PET/CT should be interpreted as part of the diagnostic pathway: it can localize active disease and detect potential biopsy targets, but incidental findings require verification and should not replace comprehensive tissue-based diagnosis. The completed CARE Checklist is available as online supplementary material (for all online suppl. material, see https://doi.org/10.1159/cro/abzag035).

## Conclusion

This case illustrates that isolated cervical lymph node metastasis may be the sole manifestation of CUP. The main practical lesson is that tissue adequacy is decisive: early escalation to excisional biopsy should be considered after an inconclusive core biopsy, particularly in poorly differentiated tumors. 18F-FDG PET/CT is valuable for orienting diagnostics, but its findings must be integrated with endoscopic evaluation, cytology, histopathology, and immunohistochemistry.

## Acknowledgments

The authors used ChatGPT (OpenAI; GPT-5.5 Thinking) for language editing and structural refinement of selected manuscript sections. No artificial intelligence tool was used to generate clinical data, histopathological findings, imaging results, figures, or conclusions. All content was critically reviewed and approved by the authors, who take full responsibility for the final manuscript.

## Statement of Ethics

Written informed consent was obtained from the patient for publication of the details of this medical case and any accompanying images. Ethical approval was not required for this case report in accordance with local/national guidelines.

## Conflict of Interest Statement

The authors have no conflicts of interest to declare.

## Funding Sources

This study was not supported by any sponsor or funder.

## Author Contributions

Dorota Szydlarska: conception and design of the case report, clinical supervision, critical revision of the manuscript, and final approval of the manuscript. Zuzanna Banaszkiewicz: data collection, literature review, manuscript drafting, preparation of the revised manuscript, correspondence with the journal, and final approval of the manuscript. Małgorzata Kołos: histopathological assessment and interpretation, pathology figure preparation and verification, critical revision of pathology-related content, and final approval of the manuscript. Alicja Jakubowska: acquisition and verification of clinical data, critical revision of the manuscript, and final approval of the manuscript. Katarzyna Życińska: academic supervision, critical revision of the manuscript for important intellectual content, and final approval of the manuscript. All authors agree to be accountable for all aspects of the work.

## Data Availability

All data generated or analyzed during this case report are included in this article and its online supplementary material. Further inquiries can be directed to the corresponding author.
